# In sickness and in health—Type I interferon and the brain

**DOI:** 10.3389/fnagi.2024.1403142

**Published:** 2024-05-07

**Authors:** Wei Cao

**Affiliations:** Department of Anesthesiology, Critical Care and Pain Medicine, McGovern Medical School, University of Texas Health Science Center at Houston, Houston, TX, United States

**Keywords:** interferon, aging, Alzheimer, tonic signaling, neuroinflammation, microglia

## Abstract

Type I interferons (IFN-I) represent a group of pleiotropic cytokines renowned for their antiviral activity and immune regulatory functions. A multitude of studies have unveiled a critical role of IFN-I in the brain, influencing various neurological processes and diseases. In this mini-review, I highlight recent findings on IFN-I’s effects on brain aging, Alzheimer’s disease (AD) progression, and central nervous system (CNS) homeostasis. The multifaceted influence of IFN-I on brain health and disease sheds light on the complex interplay between immune responses and neurological processes. Of particular interest is the cGAS-STING-IFN-I axis, which extensively participates in brain aging and various forms of neurodegeneration. Understanding the intricate role of IFN-I and its associated pathways in the CNS not only advances our comprehension of brain health and disease but also presents opportunities for developing interventions to modify the process of neurodegeneration and prevent age-related cognitive decline.

## Introduction

Recent advancements in neuroimmunology research have revealed an intricate link between the brain and the immune system, with crucial interactions occurring in both healthy and diseased states. These remarkable discoveries not only enhance our understanding of the brain’s function but also hold immense potential for developing novel therapeutic approaches for various complex neurological disorders.

Type I interferons (IFN-I) are a family of cytokines (including IFNα, IFNβ, IFNε, IFNκ, and IFNϖ) that signal through a common receptor complex named IFNα/β receptor (IFNAR) to trigger signal transduction and alter gene expression. Via the products of numerous interferon-stimulated genes (ISGs), IFN-I potently interferes with various stages of the virus infection cycle and thus is regarded as a vital innate immune defense against viral infections. In recent years, there has been a growing recognition of the intriguing roles played by IFN-I in the central nervous system (CNS) across diverse conditions. This mini-review aims to highlight key studies in this area, with a specific emphasis on Alzheimer’s disease (AD), the most prevalent form of neurodegenerative disorder. By revealing the multifaceted influence of IFN-I on brain aging, AD pathogenesis, and normal brain function, I hope to stimulate further exploration in this field and foster the development of novel therapeutic strategies for AD and other brain illnesses.

## IFN-I in brain aging

Aging significantly increases the risk of developing neurodegenerative diseases, particularly late-onset Alzheimer’s disease (LOAD), which affects more than 95% of Alzheimer’s patients and typically emerges after the age of 65. Inflammation is a central hallmark of aging, with the activation of the IFN-I pathway implicated in age-related tissue changes (Benayoun et al., 2019; Shavlakadze et al., 2019; Cao, 2022; Lopez-Otin et al., 2023). In the aging brain, structural alterations in white matter, along with volume loss and synaptic decline, coincide with progressive cognitive impairment (Radulescu et al., 2021; Bethlehem et al., 2022; Lopez-Otin et al., 2023).

Remarkably, a prominent IFN-I signature manifests in the aged mouse choroid plexus (CP), a critical structure at the blood-cerebrospinal fluid (CSF) barrier responsible for CSF production (Baruch et al., 2014). By way of selective blocking and overexpression, Michal Schwartz and colleagues showed that IFN-I negatively impacts cognitive function, likely by impeding hippocampal neurogenesis while counteracting with IFNγ, i.e., Type II IFN, within CP in aging animals (Baruch et al., 2014; Deczkowska et al., 2017). More recently, Buckley et al. (2023) developed brain “aging clocks” based on single-cell transcriptomics of the neurogenic region in the subventricular zone. Notably, they found that “Type I interferon signaling pathway” and “Cellular response to Type 1 interferon” are among the top biological processes shared across all brain cell types, underscoring the significant influence of IFN-I in brain aging.

Microglia, as brain-resident immune cells, play a vital role in maintaining brain health by providing support, nutrition, and immune defense (Paolicelli et al., 2022). Single-cell RNA sequencing (scRNAseq) of microglia isolated from aging mouse brains has revealed a subpopulation expressing ISGs, indicating IFN-I activity within the brain parenchyma (Deczkowska et al., 2017; Hammond et al., 2019). Aging triggers a myriad of molecular alterations in cells, resulting in the accumulation of senescent cells in various tissues. Activation of the innate immune cGAS-STING pathway in senescent cells leads to IFN-I production, which is a late component of the senescence-associated secretory phenotype (Yang et al., 2017; De Cecco et al., 2019). Recently, Gulen et al. (2023) identified the cGAS-STING pathway as a critical driver of chronic neuroinflammation and functional decline during brain aging. As we know, IFN-I production is typically triggered by innate immune responses to microbial or self-nucleic acids (Gilliet et al., 2008). Gulen et al. (2023) discovered that aging microglia accumulate cytosolic mitochondrial DNA, which can be recognized by STING and stimulates microglial activation and IFN-I production. Furthermore, they showed that aberrant microglial response is sufficient to trigger neurodegeneration and cognitive decline. Remarkably, STING blockade effectively attenuates age-related inflammation and improves brain function.

Collectively, these intriguing findings indicate that innate IFN-I activation is a conserved response to brain aging and exerts a detrimental effect on brain function and cognition.

## IFN-I in Alzheimer’s disease

AD, the leading cause of dementia, is characterized by progressive memory impairment, cognitive decline, and eventually the loss of ability to perform daily tasks. While the hallmark pathological features of AD include the accumulation of amyloid plaques and neurofibrillary tangles, emerging research has identified neuroinflammation as a critical contributor to disease progression (Wilson et al., 2023). Various inflammatory pathways, such as the complement cascade and inflammasome, have been implicated in the pathogenesis of AD. Leveraging a CNS-specific panel of ISGs, Roy et al. (2020) uncovered a positive correlation between the activation of IFN pathway and cognitive decline in clinical AD. Notably, the IFN-I signature was found to escalate with increased disease stage, indicating a potential link between IFN-I signaling and AD progression. Furthermore, a recent analysis of an AD cohort has revealed significantly elevated levels of IFN-β protein in the CSF of AD patients (Shen et al., 2023). These elevated IFN-β levels were found to correlate with AD core biomarkers, impaired cognitive performance, and patterns of brain atrophy. Additionally, increased signaling through the cGAS-STING pathway has been detected in human AD tissues (Udeochu et al., 2023; Xie et al., 2023). These findings collectively underscore the prominence of IFN pathway activation as a key feature of neuroinflammation in AD. Understanding its functional involvement in AD pathogenesis is thus imperative.

Remarkably, the brains of various transgenic amyloid precursor protein (APP) mouse models accumulate amyloid-beta (Aβ) plaques containing nucleic acids, which innately activate the encapsulating microglia and induce the expression of ISGs (Roy et al., 2020). Reporter-based fate mapping has revealed robust age-dependent IFN-I signaling first in microglia and then in other brain cells (Roy et al., 2022). Of note, scRNAseq studies have identified different populations of microglia based on gene expression clustering. Among these, interferon response microglia (IRM) represents a unique molecular state distinct from disease-associated microglia (DAM) or activated response microglia (ARM) (Deczkowska et al., 2018). While DAM/ARM has generally been considered neuroprotective in the presence of Aβ plaques, delineation of microglia after selective IFN-I blocking and conditional ablation of *IFN-I receptor 1* (*Ifnar1*) has revealed that microglia activated by IFN-I mediates excessive removal of synaptic structures – a functional trait confirmed by studies in other neurodegenerative conditions (Roy et al., 2022; Roy and Cao, 2022).

Due to the widespread expression of its receptors, IFN-I can profoundly affect all brain cells. Of particular note is its ability to induce the expression of interferon-induced transmembrane protein 3 (IFITM3) in neurons and astrocytes (Hur et al., 2020). This induction of IFITM3 enhances γ-secretase activity, thereby promoting the cleavage of APP and increasing amyloid pathology. Consistently, selective ablation of *Ifnar1* from neural cells reduces Aβ plaques, confirming the existence of a feedforward loop where plaque-induced inflammation exacerbates AD pathology (Roy et al., 2022). Moreover, the heightened IFN-I response to Aβ plaques stimulates neural signal transducer and activator of transcription 1 (STAT1) activity, leading to pathogenic presynaptic loss (Yasuda et al., 2021; Roy et al., 2022). Thus, IFN-I orchestrates diverse cellular changes to compromise synaptic integrity and promote Aβ pathology. Similar to findings in the aging brain, blocking IFN-I receptor signaling proves beneficial in mice with β-amyloidosis, rescuing memory and synaptic deficits while reducing microgliosis, inflammation, and neuritic pathology (Roy et al., 2020, 2022). Furthermore, Xie et al. (2023) demonstrated that *Cgas* deficiency protects AD mice from developing cognitive impairment, plaque pathology, neuroinflammation, and other AD-associated changes. In addition, intraperitoneal administration of STING inhibitor H-151 was effective in ameliorating Aβ pathology. These findings collectively underscore the pathogenic immune axis of cGAS-STING-IFN-I in promoting brain pathologies associated with β-amyloidosis.

Microtubule-associated protein tau plays a pivotal role in the clinical development of AD. Remarkably, an IFN-I signature is prevalent in both human AD and mouse brains developing tauopathy (Rexach et al., 2020; Roy et al., 2020). Interestingly, exogenous IFN-I treatment significantly potentiates seeded tau aggregation in mixed neural cultures *in vitro* (Sanford et al., 2024). Moreover, the brains of aged P301S-tau mice lacking *Ifnar1* contain markedly reduced tau pathology, indicating a pathogenic involvement of IFN-I. Recently, Udeochu et al. (2023) discovered that tau fibrils induce mitochondrial DNA leakage, cGAS-STING activation, and IFN-I response in microglia. Importantly, genetic *Cgas* ablation mitigates microglial IFN-I activity and rescues memory, synapse loss, and plasticity in a tauopathy model *in vivo*. Moreover, treatment of a novel small molecule cGAS inhibitor demonstrated efficacy against synapse loss and cognitive deficits in tauopathy mice. Hence, these reports point to the cGAS-STING-IFN-I axis as a key driver of tau pathology and associated cognitive impairment.

LOAD is influenced by various factors, including genetic predisposition, with multiple genes modifying the risk of developing the condition (Bellenguez et al., 2022). Intriguingly, Ramdhani et al. (2020) investigated disease-associated gene networks and identified IFN-I signaling pathway as trans-targets for AD risk loci. Conversely, IFN-I has been shown to functionally interact with several genetic risk factors for AD. MEF2C, encoding the Myocyte Enhancer Factor 2C transcription factor, is a candidate AD risk gene with the capacity to confer resilience to neurons against degeneration (Barker et al., 2021). Loss of MEF2C is associated with heightened IFN-I signature in microglia during aging and β-amyloidosis, while IFN-I signaling in neurons diminishes MEF2C-associated cognitive resilience in tauopathy (Deczkowska et al., 2017; Barker et al., 2021; Xue et al., 2021; Udeochu et al., 2023). Identified by genome-wide association studies, BIN1 is a leading modulator of genetic risk in AD (Bellenguez et al., 2022). Xue et al. (2021) examined microglial BIN1 function and discovered that BIN1 is a key regulator of IFN-I responses and disease-related phenotypic changes in microglia. Additionally, Apolipoprotein E4 (APOE4) gene variant represents the strongest genetic risk factor for LOAD (Bellenguez et al., 2022). Recently, Zhou et al. (2023) identified Leukocyte Immunoglobulin-Like Receptor B3 (LilrB3) as a putative cell surface receptor of APOE4. This ligand-receptor engagement induces a robust IFN-I signature in a human microglia cell line, suggesting a potential mechanism yet to be validated *in vivo*.

Overall, the convergence of evidence from genetic, molecular, and pathological studies supports the notion that aberrant IFN-I activation is closely associated with AD progression and is intertwined with genetic risk determinants of the disease.

## Tonic brain IFN-I signaling

Under homeostatic conditions, the body maintains low levels of IFN-I constitutively in peripheral tissues. The commensal microbiota in the gut, lung, and skin plays a crucial role in regulating the production of tonic IFN-I (Abt et al., 2012; Bradley et al., 2019; Di Domizio et al., 2020; Gutierrez-Merino et al., 2020; Schaupp et al., 2020; Lima-Junior et al., 2021). Such interaction between the microbiome and the immune system helps regulate the balance between protective immunity and tolerance to harmless microorganisms and environmental stimuli. Tonic IFN-I signaling also contributes to the overall surveillance of the immune system, priming it to respond rapidly and effectively to potential threats such as viral infections (Gough et al., 2012; Bradley et al., 2019; Marie et al., 2021). Moreover, it aids in the maintenance of tissue integrity and barrier function, further bolstering the body’s defenses against pathogens.

Recent research has shed light on the molecular mechanism underlying the tonic IFN-I signaling in the CNS. The brain, much like peripheral tissues, maintains basal levels of IFN-I, which enables it to defend against opportunistic infections (Owens et al., 2014; Swanson and McGavern, 2015). A study by Dorrity et al. (2023) revealed that human embryonic stem cell (hESC)-derived neurons possess the capability to spontaneously express a panel of ISGs due to an innate immune response to exceptionally high levels of double-stranded RNAs (dsRNAs) that are intrinsically accumulated. Specifically, these neurons preferentially express long 3′ untranslated regions, which give rise to immunogenic dsRNA structures that are recognized by the dsRNA-sensing machinery within the neurons, leading to the activation of IFN-I signaling. Importantly, this process is tightly regulated by ADAR1, a dsRNA-editing enzyme, as loss of ADAR1 function results in increased levels of dsRNA, heightened production of IFN-β, and eventual neuronal death. This dysregulated state mirrors the characteristics of cerebral interferonopathy, a condition associated with excessive IFN-I activation in the brain (Crow and Stetson, 2022).

Moreover, recent research has unveiled a broader role for tonic IFN-I signaling in the brain, extending beyond its classical function in host protection. Ejlerskov et al. (2015) made a groundbreaking discovery by revealing a novel protective role for IFN-β in neuronal homeostasis. They found that mice lacking IFN-β signaling specifically in neurons spontaneously develop protein aggregation in the brain due to defects in autophagy, resulting in dopaminergic neuronal loss. Conversely, low doses of recombinant IFN-β were shown to promote autophagy flux and clearance of α-synuclein, a protein associated with neurodegenerative diseases, in cultured cortical neurons. Furthermore, Hosseini et al. (2020) demonstrated that baseline levels of IFN-I signaling are necessary for normal hippocampal synaptic plasticity. By selectively ablating the *Ifnar1* gene in specific cell types, they found that IFN-I signaling in astrocytes, rather than neurons, is essential for normal spatial learning and memory formation. This effect is likely mediated by the modulation of astrocytic glutamate–aspartate transporter levels.

These studies highlight the underappreciated functional impact of tonic IFN-I signaling in maintaining brain homeostasis and cognitive function. Beyond its part in host defense, IFN-I signaling appears to play a crucial role in regulating neuronal proteostasis, synaptic plasticity, and cognitive capacity.

## Discussion

**Figure 1:**
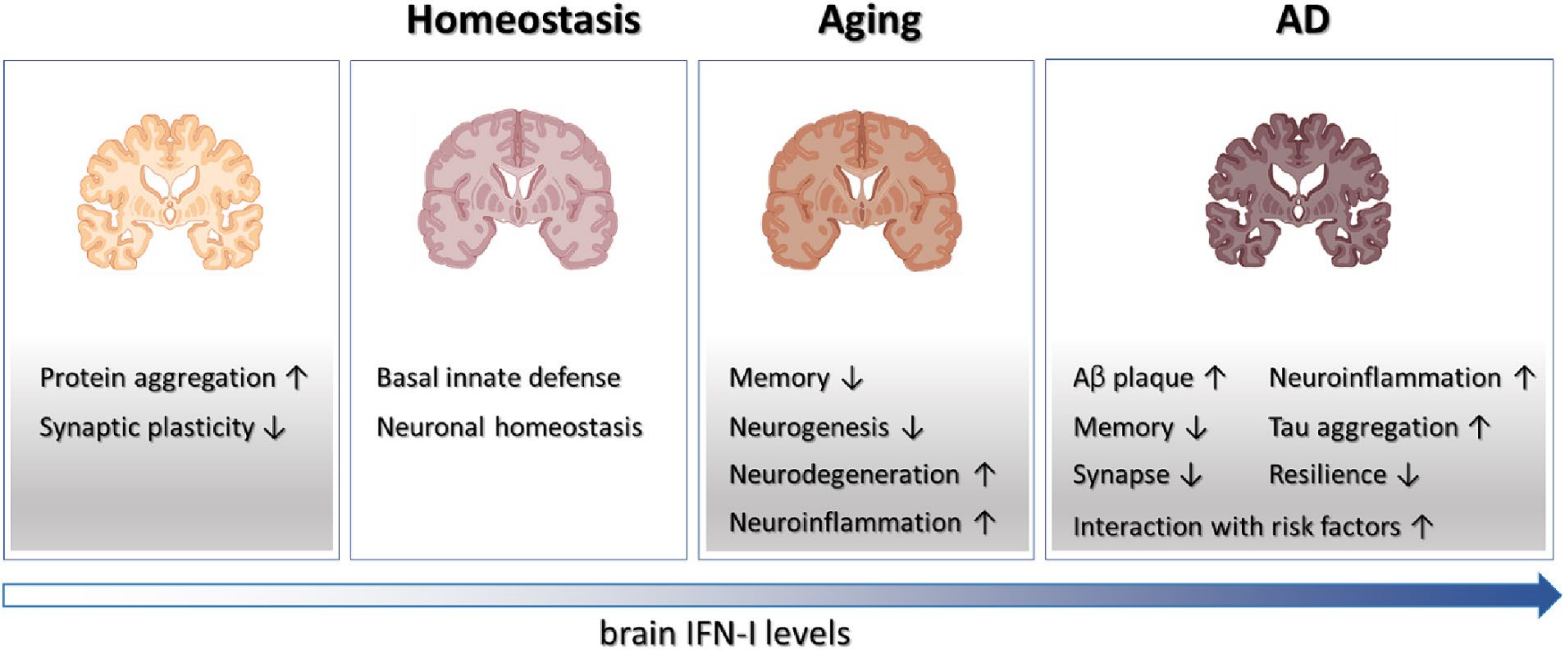
Schematics on the roles played by IFN-I in brain physiology and diseases. Tonic IFN-I signaling in healthy brain conveys protection against viral infections and maintains neuronal homeostasis. However, IFN-I deficiency results in protein aggregation and defective synaptic plasticity, while elevated IFN-I levels in aging and AD brain contribute to various aspects of degenerative processes.

Historically, the adverse effects of IFN-I on cognition and neuropsychiatric functions were first observed in patients undergoing recombinant IFN-I therapies (Valentine et al., 1998; Asnis and de la Garza, 2005). These treatments, initially used for conditions like viral infections, multiple sclerosis, and certain cancers, showed detrimental impacts on cognitive function and mental well-being in some individuals. Moreover, research on Type I interferonopathy, a group of autoinflammatory disorders characterized by dysregulated IFN-I signaling, has highlighted the damaging influence of excessive IFN-I activity on brain structure and function (Crow and Stetson, 2022). Recent studies, as reviewed here, have expanded our understanding, revealing the involvement of aberrant IFN-I activation in neurodegeneration and brain aging (Figure 1). This broader perspective underscores the significant role of this innate immune cytokine in various brain pathologies. However, alongside these negative effects, there’s growing recognition of IFN-I’s beneficial role in maintaining normal brain function. This dual nature of IFN-I—both detrimental and beneficial—presents intriguing opportunities for further investigation and translational research.

Understanding cell type-specific functions of IFN-I during neurodegeneration is a crucial area for further research. While microglia have been extensively studied in this context, recent evidence suggests that astrocytes and brain endothelial cells also undergo IFN-I activation within AD brains (Roy et al., 2022). Interestingly, cell-autonomous activation of antiviral pathways in astrocytes has been detected in models with TDP-43 pathology (Licht-Murava et al., 2023). Such activation leads to the production of IFN-I-induced chemokines, contributing to the pathogenic process. In addition, Jana et al. (2022) showed that IFN-I treatment to brain endothelial cells compromises the integrity of their barrier function by reducing levels of adherent and tight junction proteins. Thus, heightened IFN-I signaling in brain endothelial cells from transgenic AD mouse model may adversely affect the function of blood–brain barrier (BBB) (Jana et al., 2022). Furthermore, a recent study has identified IFN-I imprinted CD8 T cells inside the aging AD mouse brains, suggesting a broader impact of IFN-I dysregulation in AD pathogenesis (Fernando et al., 2023). Overall, there remains much to be discovered about the wide-ranging actions by which IFN-I contributes to neurodegeneration, particularly in terms of its effects on different cell types within the brain.

The functional implications of the cGAS-STING and IFN pathways in various brain pathologies have been increasingly recognized (Sliter et al., 2018; McCauley et al., 2020; Yu et al., 2020; Roy and Cao, 2022). However, there is a gap in understanding the specific contributions of cGAS and STING in different brain cell types, as many studies have focused on analyzing microglia from germline knockout mice for *Cgas* or *Tmem173* (gene encoding STING) or upon systemic inhibition of cGAS or STING. In sensory neurons, the STING-IFN-I signaling axis has been identified as a critical regulator of physiological pain perception, highlighting a neuron-specific role for this pathway (Donnelly et al., 2021). Separately, neurons experiencing DNA double-strand breaks in the CK-p25 mouse model of neurodegeneration display significant activation of antiviral immune pathways (Welch et al., 2022). This finding suggests a potential link between DNA damage and innate immune responses in neurons, although the exact involvement of cGAS-STING or other nucleic acid-sensing pathways in mediating such IFN-I response in AD remains to be elucidated. The accumulation of DNA damage is a common feature of neurodegenerative diseases, raising the question about how nucleic acid-sensing-IFN-I pathways directly participate in neurodegeneration. Further research focusing on cell type-specific functions of cGAS and STING, via genetic or molecular approaches, will be essential for a better mechanistic understanding.

The cGAS-STING-IFN-I axis in the brain has emerged as a significant contributor to the pathogenesis of various neurodegenerative diseases, including AD, Parkinson’s disease, frontotemporal dementia, amyotrophic lateral sclerosis, and Down syndrome (Sliter et al., 2018; McCauley et al., 2020; Yu et al., 2020; Roy and Cao, 2022). This broad involvement makes it an attractive target for therapeutic intervention across a range of CNS degenerative conditions. However, direct targeting peripheral IFN-I poses challenges, particularly in the context of compromising the antiviral immunity of elderly individuals who are most vulnerable to viral infections such as COVID-19 and influenza. Therefore, alternative approaches are being explored. Recently, *Viengkhou* et al. demonstrated the efficacy of antisense oligonucleotides (ASO) targeting murine *Ifnar1* in reducing cerebral IFN-I signatures, neuroinflammation, and neuronal damage in a transgenic mouse model of cerebral interferonopathy (Viengkhou et al., 2024). This ASO approach offers a promising avenue for patients with aberrant brain IFN-I activity. Additionally, several small molecule inhibitors targeting STING and cGAS have been developed, some of which exhibit decent BBB accessibility. These inhibitors hold potential for therapeutic development in CNS degenerative diseases (Huang et al., 2023). Thus, it is conceivable that this area of research stays active to translate the knowledge from promising preclinical findings. As mechanistic studies continue to uncover novel pathogenic mediators within the cGAS-STING-IFN-I signaling pathway, diverse drug targets will likely be explored in the years to come, offering hope for the development of effective treatments for neurodegenerative diseases.

## Author contributions

WC: Conceptualization, Funding acquisition, Resources, Visualization, Writing – original draft, Writing – review & editing.
